# {4-[5-(4-*tert*-Butyl­phen­yl)-1,3,4-oxa­diazol-2-yl]phen­yl}methanol

**DOI:** 10.1107/S1600536810052967

**Published:** 2011-01-08

**Authors:** Yu-Ling Zhao, Zhe Lv, Bin Tian, Zhe Hou, Zhong-Rong Geng

**Affiliations:** a School of Chemical and Biological Engineering, Lanzhou Jiaotong University, Lanzhou 730070, People’s Republic of China; b Key Laboratory of Opto-Electronic Technology and Intelligent Control, Ministry of Education, Lanzhou Jiaotong University, Lanzhou 730070, People’s Republic of China

## Abstract

In the title compound, C_19_H_20_N_2_O_2_, the 1,3,4-oxadiazole ring is almost coplanar with the two neighboring benzene rings [dihedral angles = 3.76 (4) and 5.49 (4)°]. In the crystal, mol­ecules are connected by strong inter­molecular O—H⋯N hydrogen bonds, forming chains parallel to the *c* axis.

## Related literature

For the properties and applications of 1,3,4-oxadiazole derivatives, see: Hughes & Bryce (2005 ▶); Kim & Lee (2007 ▶); Kulkarni *et al.* (2004 ▶); Liang *et al.* (2003 ▶); Liou *et al.* (2006 ▶); Strukelj *et al.* (1995 ▶). For the biological activity of compounds containing the 1,3,4-oxadiazole moiety, see: Cacic *et al.* (2006 ▶); Mansour *et al.* (2003 ▶); Yar *et al.* (2007 ▶); Zhang *et al.* (2007 ▶). For synthesis of the inter­mediate, see Mashraqui *et al.* (2007 ▶).
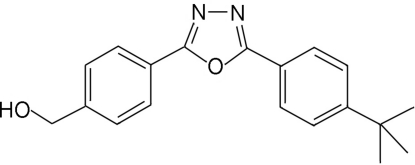


## Experimental

### 

#### Crystal data

C_19_H_20_N_2_O_2_
*M* *_r_* = 308.37
Monoclinic, 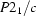

*a* = 16.3958 (18) Å
*b* = 6.0654 (7) Å
*c* = 16.7206 (19) Å
β = 102.289 (2)°
*V* = 1624.7 (3) Å^3^
*Z* = 4
Mo *K*α radiation
μ = 0.08 mm^−1^
*T* = 185 K
0.32 × 0.14 × 0.09 mm

#### Data collection

Bruker SMART APEX CCD diffractometer
Absorption correction: multi-scan (*SADABS*; Sheldrick, 1996 ▶) *T*_min_ = 0.974, *T*_max_ = 0.993
8995 measured reflections
2886 independent reflections
1805 reflections with *I* > 2σ(*I*)
*R*_int_ = 0.051

#### Refinement

*R*[*F* ^2^ > 2σ(*F* ^2^)] = 0.049
*wR*(*F* ^2^) = 0.122
*S* = 0.98
2886 reflections
212 parameters
H-atom parameters constrained
Δρ_max_ = 0.16 e Å^−3^
Δρ_min_ = −0.14 e Å^−3^

### 

Data collection: *SMART* (Bruker, 2007 ▶); cell refinement: *SAINT* (Bruker, 2007 ▶); data reduction: *SAINT*; program(s) used to solve structure: *SHELXS97* (Sheldrick, 2008 ▶); program(s) used to refine structure: *SHELXL97* (Sheldrick, 2008 ▶); molecular graphics: *SHELXTL* (Sheldrick, 2008 ▶); software used to prepare material for publication: *SHELXTL*.

## Supplementary Material

Crystal structure: contains datablocks global, I. DOI: 10.1107/S1600536810052967/pk2290sup1.cif

Structure factors: contains datablocks I. DOI: 10.1107/S1600536810052967/pk2290Isup2.hkl

Additional supplementary materials:  crystallographic information; 3D view; checkCIF report

## Figures and Tables

**Table 1:** Hydrogen-bond geometry (Å, °)

*D*—H⋯*A*	*D*—H	H⋯*A*	*D*⋯*A*	*D*—H⋯*A*
O1—H1⋯N2^i^	0.84	2.07	2.906 (3)	179

Symmetry code: (i) 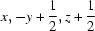.

**Fig. 1.**
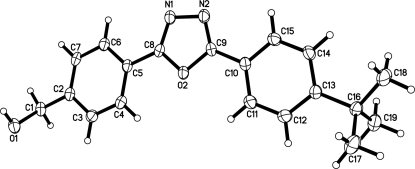
The molecule structure of the title compound. Displacement ellipsoids are drawn at the 30% probability level.

**Fig. 2.**
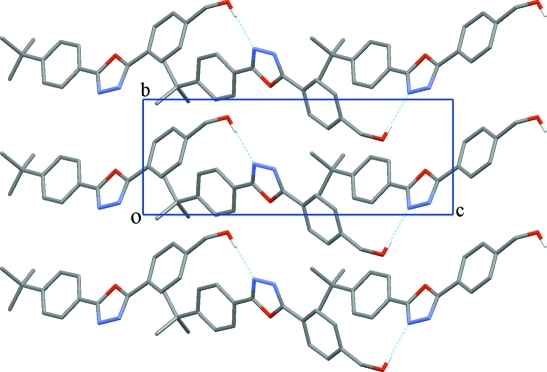
Packing diagram of the title compound showing the bc plane. Intermolecular O—H···N hydrogen bonds (dashed lines) form chains parallel to the c axis

## supplementary crystallographic information

### Comment

It is well known that 1,3,4-oxadiazole derivatives have strong electron affinity and possess electron-transporting characteristics.  They have been widely used as electroluminescent materials and as electron-transport materials in organic light-emitting diodes (OLEDs) (Strukelj *et al.*, 1995; Kulkarni *et al.*, 2004; Hughes & Bryce 2005). Due to its excellent electron transporting properties, the 1,3,4-oxadiazole unit has been embedded in larger compounds to improve the quantum efficiencies of OLEDs (Liang *et al.*, 2003; Liou *et al.*, 2006; Kim & Lee 2007). Moreover, the compounds containing 1,3,4-oxadiazole also exhibit beneficial biological activity, such as anti-inflammatory, antibacterial, anticancer, plant growth regulation, weed and worm killing, anti-HIV and other activities (Cacic *et al.*, 2006; Mansour *et al.*, 2003; Zhang *et al.*, 2007; Yar *et al.*, 2007).

The molecular structure of the title compound is shown in Fig.1. Bond lengths and angles in the molecule are within normal ranges. The 1,3,4-oxadiazole ring is almost coplanar with two neighboring benzene rings (dihedral angles between the 1,3,4-oxadiazole ring and two benzene rings are 3.76 (4)° and 5.49 (4)°. The crystal structure is stabilized by intermolecular O—H···N hydrogen bonds (Fig. 2), to form chains parallel to the *c* axis.

### Experimental

The title compound was obtained by reacting 2-[4-(bromomethyl)phenyl]-5-(4-*tert*-butylphenyl)-1,3,4-oxadiazole and potassium hydroxide in *N*,*N*-dimethyllformamide. The intermediate, 2-[4-(bromomethyl)phenyl]-5-(4-*tert*-butylphenyl)-1,3,4-oxadiazole, was synthesized according to the method described by Mashraqui *et al.* (Mashraqui *et al.*, 2007).

After dispersing potassium hydroxide (3.62 mmol) in *N*,*N*-dimethylformamide (30 ml) for 10 min, 2-[4-(bromomethyl)phenyl]-5-(4-*tert*-butylphenyl)- 1,3,4-oxadiazole (3.62 mmol) was added. The reaction vessel was refluxed for one day. Neutralization with saturated aqueous ammonium chloride (100 ml) followed by extraction with dichloromethane (100 ml) was performed, and the organic layer was dried over anhydrous magnesium sulfate. The concentrated crude product was purified with silica column chromatography to afford the title compound. m.p. 381–383 K. ^1^H-NMR (500 MHz, CDCl_3_): 8.12–8.05 (m, 4H, Ar—H), 7.56–7.52 (m, 4H, Ar—H), 4.81 (s, 2H, –CH_2_–), 2.31 (s, 1H, –OH), 1.41 (s, 9H, –CH_3_).

Colourless single crystals were obtained by slow evaporation of a methanolic solution at room temperature.

### Refinement

H atoms were positioned geometrically and refined as riding atoms, with C—H = 0.95 (aromatic), 0.99 (CH_2_), 0.98 Å (CH_3_) and *U*_iso_(H) = 1.2(1.5 for methyl)*U*_eq_(C). The hydroxyl H atom was found in a difference Fourier map and refined as riding atom, with O—H = 0.84 Å and *U*_iso_(H) = 1.5*U*_eq_(O).

### Crystal data

**Table:**

C_19_H_20_N_2_O_2_	*F*(000) = 656
*M**_r_* = 308.37	*D*_x_ = 1.261 Mg m^−^^3^
Monoclinic, *P*2_1_/*c*	Melting point = 381–383 K
Hall symbol: -P 2ybc	Mo *K*α radiation, λ = 0.71073 Å
*a* = 16.3958 (18) Å	Cell parameters from 1372 reflections
*b* = 6.0654 (7) Å	θ = 2.5–23.3°
*c* = 16.7206 (19) Å	µ = 0.08 mm^−^^1^
β = 102.289 (2)°	*T* = 185 K
*V* = 1624.7 (3) Å^3^	Block, colorless
*Z* = 4	0.32 × 0.14 × 0.09 mm

### Data collection

**Table:**

Bruker SMART APEX CCD diffractometer	2886 independent reflections
Radiation source: sealed tube	1805 reflections with *I* > 2σ(*I*)
graphite	*R*_int_ = 0.051
φ and ω scans	θ_max_ = 25.1°, θ_min_ = 2.5°
Absorption correction: multi-scan (*SADABS*; Sheldrick, 1996)	*h* = −14→19
*T*_min_ = 0.974, *T*_max_ = 0.993	*k* = −7→7
8995 measured reflections	*l* = −19→19

### Refinement

**Table:**

Refinement on *F*^2^	Primary atom site location: structure-invariant direct methods
Least-squares matrix: full	Secondary atom site location: difference Fourier map
*R*[*F*^2^ > 2σ(*F*^2^)] = 0.049	Hydrogen site location: inferred from neighbouring sites
*wR*(*F*^2^) = 0.122	H-atom parameters constrained
*S* = 0.98	*w* = 1/[σ^2^(*F*_o_^2^) + (0.0608*P*)^2^] where *P* = (*F*_o_^2^ + 2*F*_c_^2^)/3
2886 reflections	(Δ/σ)_max_ < 0.001
212 parameters	Δρ_max_ = 0.16 e Å^−^^3^
0 restraints	Δρ_min_ = −0.14 e Å^−^^3^

### Special details

**Table:**

Geometry. All e.s.d.'s (except the e.s.d. in the dihedral angle between two l.s. planes) are estimated using the full covariance matrix. The cell e.s.d.'s are taken into account individually in the estimation of e.s.d.'s in distances, angles and torsion angles; correlations between e.s.d.'s in cell parameters are only used when they are defined by crystal symmetry. An approximate (isotropic) treatment of cell e.s.d.'s is used for estimating e.s.d.'s involving l.s. planes.
Refinement. Refinement of *F*^2^ against ALL reflections. The weighted *R*-factor *wR* and goodness of fit *S* are based on *F*^2^, conventional *R*-factors *R* are based on *F*, with *F* set to zero for negative *F*^2^. The threshold expression of *F*^2^ > σ(*F*^2^) is used only for calculating *R*-factors(gt) *etc*. and is not relevant to the choice of reflections for refinement. *R*-factors based on *F*^2^ are statistically about twice as large as those based on *F*, and *R*- factors based on ALL data will be even larger.

### Fractional atomic coordinates and isotropic or equivalent isotropic displacement parameters (Å^2^)

**Table:**

	*x*	*y*	*z*	*U*_iso_*/*U*_eq_	
O1	0.11403 (11)	−0.3479 (3)	0.77257 (10)	0.0481 (5)	
H1	0.1306	−0.2304	0.7974	0.072*	
O2	0.21110 (9)	0.1173 (2)	0.41106 (9)	0.0363 (4)	
N1	0.12828 (12)	0.3966 (3)	0.42308 (11)	0.0396 (5)	
N2	0.16872 (13)	0.4399 (3)	0.35861 (11)	0.0405 (5)	
C1	0.04959 (16)	−0.2996 (4)	0.70351 (14)	0.0423 (6)	
H1A	0.0045	−0.2187	0.7219	0.051*	
H1B	0.0259	−0.4397	0.6784	0.051*	
C2	0.07960 (15)	−0.1632 (4)	0.63943 (13)	0.0358 (6)	
C3	0.13751 (15)	−0.2496 (4)	0.59808 (14)	0.0388 (6)	
H3	0.1603	−0.3919	0.6119	0.047*	
C4	0.16207 (15)	−0.1294 (4)	0.53697 (13)	0.0375 (6)	
H4	0.2006	−0.1915	0.5081	0.045*	
C5	0.13098 (15)	0.0811 (3)	0.51743 (13)	0.0334 (6)	
C6	0.07323 (15)	0.1692 (4)	0.55888 (13)	0.0373 (6)	
H6	0.0512	0.3127	0.5458	0.045*	
C7	0.04808 (15)	0.0471 (4)	0.61911 (13)	0.0386 (6)	
H7	0.0086	0.1077	0.6471	0.046*	
C8	0.15439 (14)	0.2058 (4)	0.45132 (13)	0.0335 (6)	
C9	0.21555 (15)	0.2722 (4)	0.35340 (13)	0.0346 (6)	
C10	0.26874 (14)	0.2326 (4)	0.29488 (13)	0.0338 (6)	
C11	0.30966 (17)	0.0348 (4)	0.29253 (16)	0.0514 (7)	
H11	0.3055	−0.0775	0.3311	0.062*	
C12	0.35681 (17)	−0.0006 (4)	0.23410 (16)	0.0546 (8)	
H12	0.3847	−0.1377	0.2336	0.066*	
C13	0.36454 (15)	0.1577 (4)	0.17643 (14)	0.0366 (6)	
C14	0.32332 (15)	0.3538 (4)	0.18029 (14)	0.0422 (6)	
H14	0.3276	0.4666	0.1420	0.051*	
C15	0.27591 (15)	0.3921 (4)	0.23804 (14)	0.0424 (6)	
H15	0.2481	0.5293	0.2386	0.051*	
C16	0.41390 (15)	0.1098 (4)	0.10994 (14)	0.0393 (6)	
C17	0.49387 (17)	−0.0160 (5)	0.14583 (17)	0.0589 (8)	
H17A	0.5287	0.0726	0.1890	0.088*	
H17B	0.5245	−0.0455	0.1026	0.088*	
H17C	0.4798	−0.1559	0.1689	0.088*	
C18	0.4366 (2)	0.3210 (4)	0.06978 (19)	0.0703 (10)	
H18A	0.4693	0.4174	0.1117	0.105*	
H18B	0.3854	0.3971	0.0427	0.105*	
H18C	0.4696	0.2839	0.0292	0.105*	
C19	0.35905 (17)	−0.0327 (4)	0.04458 (15)	0.0532 (7)	
H19A	0.3890	−0.0653	0.0011	0.080*	
H19B	0.3074	0.0466	0.0214	0.080*	
H19C	0.3456	−0.1708	0.0693	0.080*	

### Atomic displacement parameters (Å^2^)

**Table:**

	*U*^11^	*U*^22^	*U*^33^	*U*^12^	*U*^13^	*U*^23^
O1	0.0561 (13)	0.0525 (10)	0.0353 (10)	−0.0063 (9)	0.0085 (9)	0.0032 (8)
O2	0.0408 (11)	0.0420 (9)	0.0285 (9)	0.0023 (7)	0.0127 (8)	0.0020 (7)
N1	0.0450 (14)	0.0458 (12)	0.0301 (11)	0.0014 (10)	0.0128 (10)	0.0014 (9)
N2	0.0427 (13)	0.0494 (12)	0.0323 (12)	0.0062 (10)	0.0144 (10)	0.0039 (9)
C1	0.0477 (18)	0.0502 (15)	0.0292 (14)	−0.0063 (12)	0.0088 (13)	−0.0010 (11)
C2	0.0388 (16)	0.0416 (14)	0.0275 (13)	−0.0080 (11)	0.0084 (11)	−0.0043 (10)
C3	0.0436 (16)	0.0367 (13)	0.0373 (14)	−0.0020 (11)	0.0114 (13)	−0.0019 (11)
C4	0.0393 (16)	0.0422 (13)	0.0340 (13)	−0.0038 (11)	0.0146 (12)	−0.0061 (11)
C5	0.0374 (15)	0.0375 (13)	0.0252 (12)	−0.0056 (11)	0.0067 (11)	−0.0041 (10)
C6	0.0421 (16)	0.0405 (13)	0.0298 (13)	−0.0031 (11)	0.0090 (12)	−0.0046 (10)
C7	0.0427 (16)	0.0475 (14)	0.0282 (13)	−0.0042 (12)	0.0134 (12)	−0.0102 (11)
C8	0.0345 (15)	0.0411 (13)	0.0252 (13)	−0.0011 (11)	0.0074 (11)	−0.0064 (10)
C9	0.0385 (16)	0.0407 (13)	0.0255 (13)	−0.0017 (12)	0.0085 (12)	0.0040 (10)
C10	0.0326 (15)	0.0410 (13)	0.0281 (13)	0.0004 (11)	0.0068 (11)	0.0021 (10)
C11	0.067 (2)	0.0444 (15)	0.0515 (17)	0.0111 (13)	0.0313 (16)	0.0150 (12)
C12	0.071 (2)	0.0421 (14)	0.0606 (19)	0.0167 (14)	0.0354 (17)	0.0115 (13)
C13	0.0366 (15)	0.0410 (13)	0.0342 (14)	−0.0008 (11)	0.0119 (12)	0.0010 (11)
C14	0.0444 (17)	0.0446 (14)	0.0416 (15)	0.0044 (12)	0.0180 (13)	0.0120 (12)
C15	0.0427 (16)	0.0417 (13)	0.0456 (15)	0.0108 (12)	0.0156 (13)	0.0097 (12)
C16	0.0382 (16)	0.0424 (13)	0.0411 (14)	0.0012 (12)	0.0166 (13)	−0.0005 (11)
C17	0.0458 (19)	0.0787 (19)	0.0555 (18)	0.0067 (15)	0.0181 (15)	−0.0091 (15)
C18	0.096 (3)	0.0512 (17)	0.085 (2)	−0.0044 (16)	0.066 (2)	0.0018 (15)
C19	0.0552 (19)	0.0661 (18)	0.0408 (16)	0.0017 (14)	0.0158 (14)	−0.0035 (13)

### Geometric parameters (Å, °)

**Table:**

O1—C1	1.420 (3)	C10—C15	1.379 (3)
O1—H1	0.8400	C11—C12	1.386 (3)
O2—C9	1.359 (2)	C11—H11	0.9500
O2—C8	1.368 (2)	C12—C13	1.386 (3)
N1—C8	1.288 (3)	C12—H12	0.9500
N1—N2	1.405 (2)	C13—C14	1.377 (3)
N2—C9	1.289 (3)	C13—C16	1.536 (3)
C1—C2	1.516 (3)	C14—C15	1.382 (3)
C1—H1A	0.9900	C14—H14	0.9500
C1—H1B	0.9900	C15—H15	0.9500
C2—C7	1.391 (3)	C16—C17	1.525 (3)
C2—C3	1.391 (3)	C16—C19	1.527 (3)
C3—C4	1.383 (3)	C16—C18	1.528 (3)
C3—H3	0.9500	C17—H17A	0.9800
C4—C5	1.388 (3)	C17—H17B	0.9800
C4—H4	0.9500	C17—H17C	0.9800
C5—C6	1.394 (3)	C18—H18A	0.9800
C5—C8	1.457 (3)	C18—H18B	0.9800
C6—C7	1.382 (3)	C18—H18C	0.9800
C6—H6	0.9500	C19—H19A	0.9800
C7—H7	0.9500	C19—H19B	0.9800
C9—C10	1.463 (3)	C19—H19C	0.9800
C10—C11	1.379 (3)		
			
C1—O1—H1	109.5	C10—C11—H11	119.9
C9—O2—C8	102.87 (16)	C12—C11—H11	119.9
C8—N1—N2	105.96 (17)	C11—C12—C13	122.1 (2)
C9—N2—N1	106.82 (17)	C11—C12—H12	119.0
O1—C1—C2	113.0 (2)	C13—C12—H12	119.0
O1—C1—H1A	109.0	C14—C13—C12	116.6 (2)
C2—C1—H1A	109.0	C14—C13—C16	122.48 (19)
O1—C1—H1B	109.0	C12—C13—C16	120.9 (2)
C2—C1—H1B	109.0	C13—C14—C15	122.2 (2)
H1A—C1—H1B	107.8	C13—C14—H14	118.9
C7—C2—C3	118.78 (19)	C15—C14—H14	118.9
C7—C2—C1	120.89 (19)	C10—C15—C14	120.4 (2)
C3—C2—C1	120.3 (2)	C10—C15—H15	119.8
C4—C3—C2	120.4 (2)	C14—C15—H15	119.8
C4—C3—H3	119.8	C17—C16—C19	108.9 (2)
C2—C3—H3	119.8	C17—C16—C18	108.8 (2)
C3—C4—C5	120.6 (2)	C19—C16—C18	108.7 (2)
C3—C4—H4	119.7	C17—C16—C13	110.55 (19)
C5—C4—H4	119.7	C19—C16—C13	107.72 (18)
C4—C5—C6	119.35 (19)	C18—C16—C13	111.99 (18)
C4—C5—C8	120.90 (19)	C16—C17—H17A	109.5
C6—C5—C8	119.7 (2)	C16—C17—H17B	109.5
C7—C6—C5	119.8 (2)	H17A—C17—H17B	109.5
C7—C6—H6	120.1	C16—C17—H17C	109.5
C5—C6—H6	120.1	H17A—C17—H17C	109.5
C6—C7—C2	121.1 (2)	H17B—C17—H17C	109.5
C6—C7—H7	119.4	C16—C18—H18A	109.5
C2—C7—H7	119.4	C16—C18—H18B	109.5
N1—C8—O2	112.30 (18)	H18A—C18—H18B	109.5
N1—C8—C5	128.60 (19)	C16—C18—H18C	109.5
O2—C8—C5	119.09 (19)	H18A—C18—H18C	109.5
N2—C9—O2	112.03 (17)	H18B—C18—H18C	109.5
N2—C9—C10	128.56 (19)	C16—C19—H19A	109.5
O2—C9—C10	119.41 (19)	C16—C19—H19B	109.5
C11—C10—C15	118.5 (2)	H19A—C19—H19B	109.5
C11—C10—C9	121.64 (19)	C16—C19—H19C	109.5
C15—C10—C9	119.8 (2)	H19A—C19—H19C	109.5
C10—C11—C12	120.2 (2)	H19B—C19—H19C	109.5

### Hydrogen-bond geometry (Å, °)

**Table:**

*D*—H···*A*	*D*—H	H···*A*	*D*···*A*	*D*—H···*A*
O1—H1···N2^i^	0.84	2.07	2.906 (3)	179

Symmetry codes: (i) *x*, −*y*+1/2, *z*+1/2.

## References

[bb1] Bruker (2007). *SMART* and *SAINT* Bruker AXS Inc., Madison, Wisconsin, USA.

[bb2] Cacic, M., Trkovnik, M., Cacic, F. & Has-Schon, E. (2006). *Molecules*, **11**, 134–147. 10.3390/11010134 PMC6148592 17962784

[bb3] Hughes, G. & Bryce, M. R. (2005). *J. Mater. Chem.* **15**, 94–107.

[bb4] Kim, J. H. & Lee, H. (2007). *Synth. Met.* **157**, 1040–1045.

[bb5] Kulkarni, A. P., Tonzola, C. J., Babel, A. & Jenekhe, S. A. (2004). *Chem. Mater.* **16**, 4556–4573.

[bb6] Liang, F. S., Zhou, Q. G., Cheng, Y. X., Wang, L. X., Ma, D. G., Jing, X. B. & Wang, F. S. (2003). *Chem. Mater.* **15**, 1935–1937.

[bb7] Liou, G. S., Hsiao, S. H., Chen, W. C. & Yen, H. J. (2006). *Macromolecules*, **39**, 6036–6045.

[bb8] Mansour, A. K., Eid, M. M. & Khalil, N. S. A. M. (2003). *Molecules*, **8**, 744–755.

[bb9] Mashraqui, S. H., Sundaram, S., Bhasikuttan, A. C., Kapoor, S. & Sapre, A. V. (2007). *Senss. Actuat. B*, **122**, 347–350.

[bb10] Sheldrick, G. M. (1996). *SADABS* University of Göttingen, Germany.

[bb11] Sheldrick, G. M. (2008). *Acta Cryst.* A**64**, 112–122. 10.1107/S0108767307043930 18156677

[bb12] Strukelj, M., Papadimitrakopoulos, F., Miller, T. M. & Rothberg, L. J. (1995). *Science*, **267**, 1969–1972. 10.1126/science.267.5206.1969 17770109

[bb13] Yar, M. S., Siddiqui, A. A. & Ali, M. A. (2007). *J. Chin. Chem. Soc.* **54**, 5–8.

[bb14] Zhang, C. R., Wang, L., Ge, Y. L. & Ju, X. L. (2007). *Chin. J. Org. Chem.* **27**, 1432–1437.

